# Body shaming and internalized weight bias as potential precursors of eating disorders in adolescents

**DOI:** 10.3389/fpsyg.2024.1356647

**Published:** 2024-02-06

**Authors:** Silvia Cerolini, Mariacarolina Vacca, Anna Zegretti, Andrea Zagaria, Caterina Lombardo

**Affiliations:** Department of Psychology, Sapienza University of Rome, Rome, Italy

**Keywords:** body shaming, weight bias, eating disorders, weight stigma, adolescents

## Abstract

**Introduction:**

Body shaming (BS) is a growing phenomenon within the school context, especially among adolescents. Recently, it has been described as an unrepeated act in which a person expresses unsolicited, mostly negative comments about an individual’s body. The targeted person perceives these comments as negative, offensive or body shame-inducing. Empirical evidence also suggests that body weight is the most common reason that youths are teased and bullied. Indeed, weight stigma, described as bias or discriminatory behaviors, attitudes, feelings, and thinking about individuals, because of their weight, can lead to weight-based discrimination and victimization. Preliminary evidence suggests that BS and weight stigma have negative effects on psychological health both in the short and long term. In the delicate stage of adolescence development and pubertal maturation, BS experiences can be highly prevalent and it can lead to adverse outcomes such as eating disorders (ED). However, prevalence data in the Italian context are still lacking.

**Methods:**

The study aims to estimate weight-related BS perceived by different sources (i.e., peers and family members) and their associations with public and internalized weight bias, body mass index (BMI), body dissatisfaction, and ED symptoms. A sample of 919 high school students (*M*_age_ = 15.97, SD = 1.58; 57.1% boys) completed a battery of self-report questionnaires assessing these variables.

**Results:**

One in four students reported experiences of weight-related BS by peers or family members. A total of 37% reported having at least one BS experience in a lifetime. Higher scores of ED symptoms, body dissatisfaction, and weight bias were reported by adolescents who experienced BS, especially females. Among overweight participants, results showed that internalized weight bias partially mediated the relationship between BS by family members and ED symptoms and fully mediated the relationship between BS by peers and ED symptoms, after controlling for age, sex and BMI.

**Discussion:**

These findings, despite their cross-sectional nature, add an important contribution to the creation of quantitative empirical evidence on the phenomenon of BS. Its role in explaining eating disorders, both alone and with the mediation of internalized weight stigma has been first proved and needs to be confirmed by longitudinal results.

## 1 Introduction

Body shaming (BS) is a growing phenomenon within the school and social media context, especially among high-school students (Gam et al., 2020; Schlüter et al., 2023). However, there is still a lack of a clear scientific definition of BS, while various definitions have spread across non-scientific communities (i.e., magazines, internet sites, social networks, etc.). Recently, an exploratory study by Schlüter et al. (2023) has tempted to fill this gap generating a shared definition of BS as unrepeated acts in which a person expresses unsolicited, mostly negative opinions/comments about an individual’s body, without necessarily intending to harm him/her. It can range from well-meant advice (e.g., medically-based advice from a family member: “You should lose some weight to prevent high blood pressure”) to malevolent insults (e.g., an anonymous online comment such as “She looks too skinny, she’s ugly!”). Essentially, the targeted person perceives these comments as negative, offensive or body shame-inducing. According to the authors (Schlüter et al., 2023), BS can occur online and offline, thus evolving into bullying or cyberbullying with repetition over time.

Individual’s body or appearance is the focus of BS, targeting not only people with overweight or obesity but also thin or underweight individuals, thus leading to consider BS an umbrella term that includes different forms of victimization (Schlüter et al., 2023). Fauzia and Rahmiaji (2019) categorized four forms of BS according to the targets of insulting: fat/thin shaming (i.e., negative comments directed to individuals with overweight/underweight), hair shaming (i.e., name-calling for excess hair on human bodies) and skin tone shaming (i.e., skin color discriminations). The most popular type of BS is fat shaming (Almas et al., 2021) since anti-fat attitudes, namely negative implicit prejudices toward individuals with overweight, are disconcertingly diffused in multiple life domains (e.g., schools, Pereda-Pereda et al., 2019; healthcare settings, Robinson, 2021). Empirical evidence suggests that according to students, parents, and teachers, body weight is the most common reason that youths are teased and bullied (Puhl et al., 2016). Indeed, weight stigma, described as bias or discriminatory behaviors, attitudes, feelings, and thinking about individuals, because of their weight, can lead to weight-based discrimination and victimization (Puhl and Lessard, 2020). Prejudices behind weight stigma are frequently encouraged by negative characteristics typically associated with obesity, such as laziness, lack of willpower or moral character, low intelligence, and attractiveness (Roberts and Polfuss, 2022; Tanas et al., 2022).

Weight-based teasing and bullying are reported as ordinary experiences among adolescents, especially for those with higher body weight (Bucchianeri et al., 2013; Puhl et al., 2013). Recent prevalence estimates indicate that around 25–50% of all teenagers report being discriminated against because of their weight (Puhl and Lessard, 2020). In particular, youngsters with overweight/obesity are 32% more likely to be verbally bullied by their peers, while secondary school students with obesity are 66% more likely to be victims of cyberbullying (Waasdorp et al., 2018; Puhl and Lessard, 2020). Consistently, further results show that BS victims in childhood have a higher body mass index (BMI) than their non-bullied peers and have a higher risk of developing obesity (Mamun et al., 2013; Takizawa et al., 2015). Longitudinal results documented that weight-based teasing is prevalent throughout adolescence (Haines et al., 2008), and may remain consistent during the transition into adulthood (Haines et al., 2013).

Considering this empirical evidence, we can certainly argue that weight stigma and body shaming can negatively impact the bio-psycho-social health of adolescents both in the short and long term. In support of this, cross-sectional findings documented links between weight-based victimization and negative health behaviors, disordered eating, and poorer emotional well-being (Puhl et al., 2017). A systematic review by Puhl and Lessard (2020) suggested long-lasting adverse effects of weight stigmatization by multiple interpersonal sources (i.e., peers, family members, and educators) on the psychological, social, academic, and physical well-being of youngsters. Consistently, meta-analytic results showed that weight stigma is positively associated with psychological distress (i.e., depression and anxiety, Alimoradi et al., 2020). Furthermore, little longitudinal evidence documented that weight-based teasing in adolescence predicted higher BMI and obesity 15 years later independently of gender (Puhl et al., 2017). Results from the same study (Puhl et al., 2017) also found a positive longitudinal association with eating disorder symptoms (i.e., binge eating, dieting, unhealthy weight control, eating to cope, poor body image, etc.) in female participants.

In the delicate stage of adolescence development and pubertal maturation, BS experiences can be highly prevalent, as previously suggested, and it can lead to adverse outcomes. Indeed, during this evolutive age physical changes often go in the direction of weight and shape gain that appear in contrast to the standards of beauty and thinness proposed by Western societies (Davison and McCabe, 2006; Wilhelm et al., 2020), However, prevalence data among the Italian context are still lacking. One previous study documented that 11.7% of adolescents with obesity report being verbally, relationally, and physically bullied because of their weight (Bacchini et al., 2015). Similarly, two studies by Guardabassi and Tomasetto (2020, 2022) in school-aged children documented the association of weight-based teasing with body dissatisfaction and eating restraint, and between weight stigma and impaired executive functions and low quality of life. Despite these early studies, evidence addressing BS and its potential role in explaining negative outcomes such as eating disorders in the Italian context is still scarce.

Our study aims to estimate BS experiences perceived by different sources (i.e., peers and family members) among Italian high-school students and their associations with eating disorder symptoms, BMI, and body dissatisfaction. Furthermore, we hypothesize that the effect of BS on psychological well-being may be mediated by the individual’s *weight bias (WB)*, defined as negative weight-related attitudes, beliefs, assumptions and judgments toward individuals who are overweight and obese (Washington, 2011). WB may also be distinguished into *public/explicit WB* (stereotypes, prejudices, and hostile attitudes toward individuals because of their weight) and *internalized WB* (self-stigma, applying these weight-based negative attitudes and stereotypes to themselves; Durso and Latner, 2008). Internalized WB can further increase the negative consequences of weight stigma and may be considered more important than the experience of stigma or weight status alone (Tanas et al., 2022). Recent results in adult samples showed that internalized WB may have an important role in explaining worse mental and physical health (Zagaria et al., 2023). Studies that deepen the relationships across these variables (BS, WB and EDs) in adolescence are still lacking, especially in the Italian context. For these reasons, we aim to test two mediation models among high-school students reporting overweight or obesity in which BS is inserted as the independent variable, eating disorders (ED) symptoms as the dependent variable and weight bias (respectively public and internalized WB) as mediator, controlling for age, sex and BMI of participants.

## 2 Materials and methods

### 2.1 Participants and procedure

A sample of 919 high school students (*M*_age_ = 15.97, SD = 1.58; 57.1% boys) were enrolled in the study during the academic year 2022–2023, from 10 secondary schools (grades 9–11) in the urban area of Rome using a convenience sampling method. After an initial networking phase, 12 eligible schools were identified through the institutional contacts of the authors and partners involved in the study. The school leaders were contacted by email with the proposal to participate in the study. In addition, telephone contacts or meetings in person explained the objectives and procedure of the study. Those who agreed to participate (response rate > 80%) involved teachers in the distribution of informed consent 2 weeks before the start of the study. In most cases, the researchers went in person to explain the study in detail to teenage students and collect informed parental consent signed by both parents or legal guardians. In addition, when completing the questionnaires, they were also asked for their consent to participate and informed that they could withdraw from the study at any time they wished. In agreement with the teachers, and with the presence of the researchers in the classroom, the students completed a battery of tests during the timetable of the school lessons. The online battery of self-report questionnaires hosted by the Qualtrics platform^1^ assessed BS, internalized and public WB, ED symptoms, body dissatisfaction and demographic information. This screening phase was the starting point of a project promoting an educational and experiential weight-related teasing/bullying prevention program, targeting the reduction of BS, weight stigma and associated negative outcomes. The protocol was approved by the Institutional Review Board of the Department of Psychology at Sapienza University of Rome (prot. Number 0001069).

### 2.2 Measures

#### 2.2.1 Sociodemographic characteristics

Participants provided information related to their age, sex (as it appeared on their birth certificate), gender identity, school and class attended. They were also asked to report anthropometrics (i.e., height and weight) to calculate their BMI and to refer if they perceived themselves as overweight (yes/no).

#### 2.2.2 Experiences of body shaming

Two yes/no questions were used to assess weight-related BS experiences from two sources: from peers (“Have you ever been teased or made fun because of your weight by peers?”) and from family members (“Have you ever been teased or made fun because of your weight by a family member”) These questions were previously employed by Puhl et al. (2017) and demonstrated good psychometric properties.

#### 2.2.3 Weight bias

The Attitudes Toward Obese Persons Scale (ATOP, Allison et al., 1991), in the Italian version validated by Zagaria et al. (2022), was used to assess perceptions and attitudes about people with obesity. The Italian version of the scale consisted of 16 items rated on a six-point Likert scale, ranging from −3 (strongly disagree) to 3 (strongly agree). The scale measures explicit attitudes or stereotypes about obesity. Examples of proposed items are: “Obese workers cannot be as successful as other workers” or “One of the worst things that could happen to a person would be for him to become obese.” As stated by the original authors of the ATOP (Allison et al., 1991; see also Tsai et al., 2019), the scoring consisted of three steps: (1) reverse coding the negatively worded items, (2) summing the scores of the items, and (3) adding 60 to the summated score. This was done to avoid negative scores, as the scoring of the ATOP items ranged from −3 to 3. Finally, lower scores indicated more negative attitudes. Both original and Italian validation studies confirmed its validity and reliability. Cronbach’s alpha in the present sample was 0.765, indicating that the scale was internally consistent.

The Weight Bias Internalization Scale (WBIS, Durso and Latner, 2008) in the Italian version by Innamorati et al. (2017) was administered to assess the internalization of WB and negative stereotypes about overweight and obesity. For this purpose, the scale was electronically available only if participants previously responded “Yes” to the item assessing whether they perceived themselves as overweight. The 11 items were rated on a 7-point Likert scale ranging from 1 (strongly disagree) to 7 (strongly agree). Once added together, higher scores indicated greater internalization of weight-related stigma. Examples of presented items are: “I do not feel that I deserve to have a fulfilling social life, as long as I’m overweight” and “My weight is a major way that I judge my value as a person.” This unidimensional scale demonstrated satisfactory convergent and criterion validity (Innamorati et al., 2017). Cronbach’s alpha in the present sample was 0.904, confirming excellent internal validity.

#### 2.2.4 Eating disorders symptoms

The Disordered Eating Questionnaire (DEQ, Lombardo et al., 2004) was used to assess disordered eating-related behaviors and attitudes. This 24-item scale allows us to calculate a valid and reliable global score of ED symptomatology (e.g., restrictive eating, binge eating and purging behaviors, willingness to lose weight, ruminating, and worrying about weight and body shape, engaging in intense physical exercise to lose weight, etc.). A clinical cutoff score of 30 has been previously demonstrated as indicative of the presence of ED symptoms (Lombardo et al., 2011). In the present study, Cronbach’s alpha was 0.935, showing excellent internal validity.

#### 2.2.5 Body size dissatisfaction

The Silhouette Rating Scale (SRS, Lombardo et al., 2022) is a pictorial tool depicting a series of nine female or male silhouettes varying in body dimensions (width of body parts) and shape. Images ranged from the thinnest (the first) to the larger (the ninth) silhouette. Two items assess current and ideal body size evaluation, allowing to estimate a score of body size dissatisfaction obtained from the discrepancy between them (ideal minus current body shape and size ratings). Negative ratings indicate more body dissatisfaction. SRS showed good psychometric properties and it guaranteed the universality of use thanks to the absence of details related to ethnicity or culture and at the same time, maintaining the right level of realism.

### 2.3 Data analyses

Data were analyzed using Jamovi 2.3 (2022). Descriptive statistics and bivariate correlations among the main variables under investigation were calculated. To assess gender differences, independent sample *t*-tests were conducted along with Cohen’s d to quantify the standardized mean difference. Similarly, independent sample *t*-tests were carried out to assess mean differences in key variables between individuals experiencing BS and those who were not. Eventually, two mediation models were computed, the first examining public WB, and the second examining internalized WB, as potential mediators of the relationship between BS and ED symptoms. Age, sex, and BMI were inserted as covariates in both models. Following MacKinnon et al. (2004), the significance of the indirect effects was formally tested by calculating 95% bias-corrected bootstrap confidence intervals (5,000 replications).

## 3 Results

### 3.1 Descriptive statistics

Descriptive statistics of the study variables are reported in Table 1. The mean BMI was 21.93 (SD = 4.23) and based on it, 15.3% of the participants were classified in a condition of overweight according to the World Health Organization (BMI ≥ 25). Similarly, a slightly higher percentage of 16.6% indicated to perceive themselves in a condition of overweight (i.e., they answered “yes” to the single item “Do you perceive yourself as overweight?”). More specifically, 86 participants who were not classified as overweight within the BMI range referred to perceived themselves as overweight, while 38 participants who described themselves as not overweight were classified as overweight according to their BMI.

**Table 1:** Descriptive statistics and bivariate correlations.

Variable	*M* (SD)	1	2	3	4	5
1. Internalized WB	42.80 (16.49)					
2. Public WB	64.01 (14.56)	−0.319**				
3. ED symptoms	28.40 (22.68)	0.736**	−0.015			
4. Body size dissatisfaction	−0.73 (1.88)	−0.519**	−0.066*	−0.587**		
5. BMI	21.93 (4.23)	0.145*	0.030	0.265**	−0.468**	

Calculations were based on the whole sample, except for internalized WB which was assessed only in a subsample of adolescents who perceived themselves in a condition of overweight. *p < 0.05; **p < 0.001.

Among all the adolescents, 25% reported having experienced BS by family members, while 25.1% experienced BS by peers. Moreover, 39.6% of the sample reported clinically significant ED symptoms (DEQ score ≥ 30).

As shown in Table 1, bivariate correlations showed that most of the examined variables significantly correlated (ranging from small to large effects). As we expected, internalized WB in participants perceiving themselves as overweight was significantly associated with all the investigated variables, especially with ED symptoms (*r* = 0.736, *p* < 0.001) and body size dissatisfaction (*r* = −0.519, *p* < 0.001). Conversely, public WB did not correlate to ED symptoms, BMI and only weakly and negatively with body size dissatisfaction (*r* = 0.066, *p* < 0.05). Finally, as we expected, BMI, ED symptoms, and body size dissatisfaction significantly correlated with large effects.

### 3.2 Sex differences

As reported in Tables 2, 3, independent sample *t*-tests revealed that females reported higher levels of internalized WB (Cohen’s d = 0.659), ED symptoms (Cohen’s d = 0.954), and body size dissatisfaction (Cohen’s d = −0.895) compared to males. The only exception was public WB, which was significantly higher in males compared to females (lower scores indicating more negative attitudes, Cohen’s d = 0.218).

**Table 2:** Independent sample *t*-tests across gender.

	Females	Males			
Variable	Mean (SD)	Mean (SD)	*t* (df)	*p*-value	Cohen’s d
Internalized WB	47.80 (15.38)	37.46 (16.01)	5.461 (273)	<0.001	0.659
Public WB	65.80 (14.05)	62.64 (14.81)	3.418 (1003)	<0.001	0.218
ED symptoms	39.51 (24.60)	19.93 (16.75)	14.779 (976)	<0.001	0.954
Body size dissatisfaction	−1.61 (1.87)	−0.07 (1.59)	−13.817 (970)	<0.001	−0.895

Internalized WB was assessed only in a subsample of adolescents who perceived themselves in a condition of overweight.

**Table 3:** Distribution of female and male participants and experiences of body shaming.

		Sex		
BS experiences		Females	Males	Total
No BS	N	201	404	605
	% row	33.2%	66.8%	100%
	% column	48.4%	74%	63%
At least one BS	N	214	142	356
	% row	60.1%	39.9%	100%
	% column	51.6%	26%	37%
Total	N	415	546	961
	% row	43.2%	56.8%	100%
	% column	100%	100%	100%

Moreover, 51.5% of the female participants reported at least one experience of BS by family members or peers, compared to 26% of male participants. In total, 37% of participants reported at least one experience of BS by family or peers. The contingency table (Table 3) shows these distributions, and the chi-square test of independence confirmed that gender and BS experiences were significantly associated (χ^2^ = 66.046, df = 1, *p* < 0.001).

### 3.3 Differences between those who experienced BS and those who did not experience BS

Table 4 shows that adolescents who reported BS experiences showed higher levels of internalized WB (Cohen’s d = −1.128), public stigma (Cohen’s d = 0.132), ED symptoms (Cohen’s d = −1.116), and body size dissatisfaction (Cohen’s d = 0.706) compared to adolescents who did not report BS experiences.

**Table 4:** Independent sample *t*-tests across BS experiences.

	Without BS experiences	With at least one BS experience			
Variable	Mean (SD)	Mean (SD)	*t* (df)	*p*-value	Cohen’s d
Internalized WB	33.21 (14.02)	49.49 (14.71)	−9-204 (273)	<0.001	−1.128
Public WB	64.69 (14.37)	62.78 (14.74)	1.969 (957)	0.049	0.132
ED symptoms	20.15 (17.65)	42.43 (23.34)	−16.710 (959)	<0.001	−1.116
Body size dissatisfaction	−0.28 (1.51)	−1.53 (2.17)	10.566 (959)	<0.001	0.706

Internalized WB was assessed only in a subsample of adolescents who perceived themselves in a condition of overweight. *p < 0.05; **p < 0.001.

### 3.4 Mediation models

**Figure 1:**
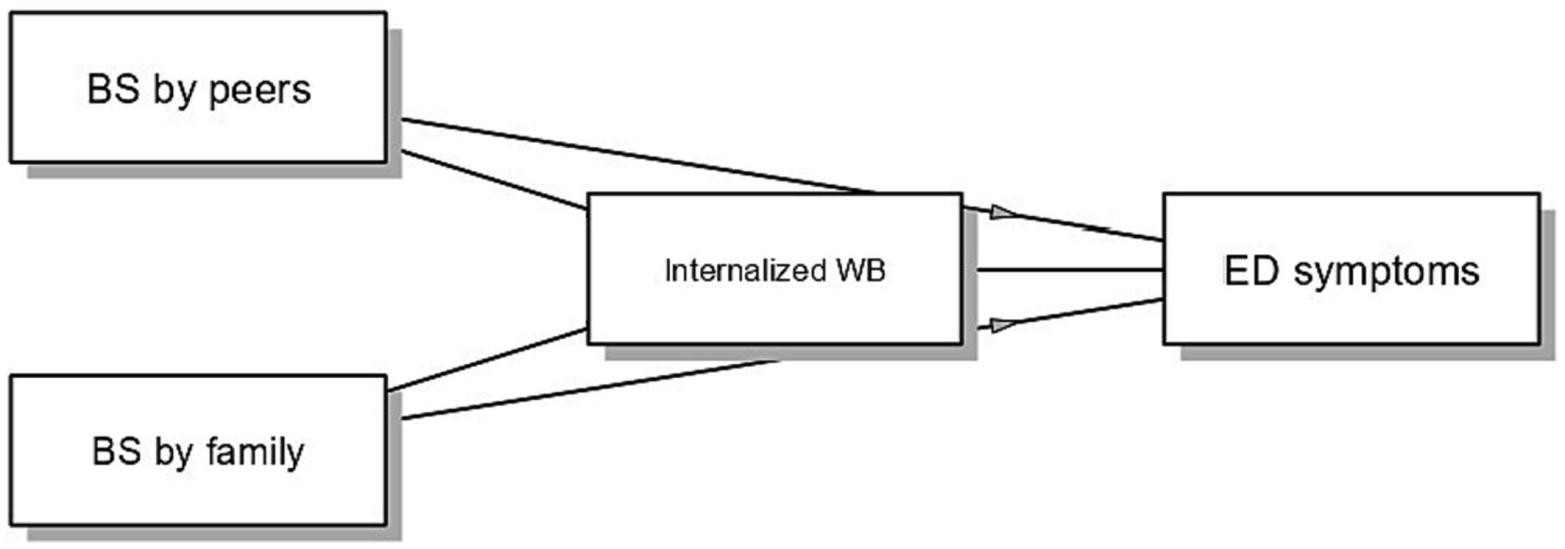
Mediation model examining the indirect relationship between BS and ED symptoms via internalized WB. The model was estimated on the subsample of adolescents who perceived themselves in a condition of overweight. BS by peers and BS by family were coded as 0 = not experienced and 1 = experienced.

In the first model, we examined the role of internalized WB as a mediator in the relationship between BS and ED symptoms (see Figure 1). Sex, age and BMI were included as covariates. Findings showed that BS by peers (*B* = 7.364, *p* < 0.001) and BS by family (*B* = 5.904, *p* = 0.008) were significantly associated with internalized WB, which in turn was positively associated with ED symptoms (*B* = 0.859, *p* < 0.001). Moreover, BS by family directly impacted ED symptoms (*B* = 5.684, *p* = 0.013), while the direct path from BS by peers was not significant (*B* = 0.656, *p* = 0.768). Importantly, internalized WB partially mediated the relationship between BS by family members and ED symptoms (*B* = 5.074, 95% BCI 1.467–9.031), and fully mediated the relationship between BS by peers and ED symptoms (*B* = 6.329, 95% BCI 3.051–10.132).

**Figure 2:**
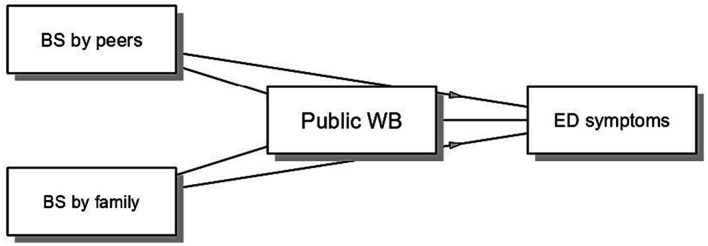
Mediation model examining the indirect relationship between BS and ED symptoms via public WB. The model was estimated on the subsample of adolescents who perceived themselves in a condition of overweight. BS by peers and BS by family were coded as 0 = not experienced and 1 = experienced.

In the second model, we assessed the role of public WB as a mediator in the relationship between BS and ED symptoms (see Figure 2). Sex, age and BMI were considered as covariates. Both BS by peers (*B* = −1.790, *p* = 0.442) and BS by family (*B* = 1.525, *p* = 0.531) did not predict public WB. Similarly, public WB was not significantly related to ED symptoms (*B* = −0.130, *p* = 0.151). Therefore, public WB did not mediate the relationship between BS by family and peers and ED symptoms (*B* = −0.198, 95% BCI −1.493 to 0.286, and *B* = 0.233, 95% BCI −0.264 to 1.651, respectively). Substantiating previous results, BS by peers (*B* = 6.991, *p* = 0.013) and BS by family (*B* = 11.157, *p* < 0.001) exerted direct effects on ED symptoms.

## 4 Discussion

This study primarily aimed to estimate the prevalence of weight-related body shaming experiences among a large sample of Italian high-school students and its associations with ED symptoms. Associations among these experiences and internalized and public weight bias, BMI, and body size dissatisfaction have been also established highlighting significant gender differences. Furthermore, we expanded previous research on the association between body shaming and ED symptoms in adolescents reporting overweight by evaluating the mediating role of internalized and public weight bias.

First, results showed that one in four high-school students reported experiencing weight-related BS by peers (25.1%) or by family members (25%), thus leading to a total percentage of 37% who reported having at least one BS experience in their lifetime. These numbers are consistent with previous synthesized results collected internationally (Puhl and Lessard, 2020) and constitute the first evidence in the Italian context that is still underrepresented through strong empirical evidence. Similarly, only Bacchini et al. (2015) estimated that 11.7% of adolescents with obesity reported being verbally, relationally, and physically bullied because of their weight, possibly underestimating these experiences. In addition, nearly 40% of participants reported clinically significant ED symptoms, which signaled an alarming growing prevalence rate, also compared to previous studies from the Italian context that documented a prevalence rate of 28.5% (e.g., Toselli et al., 2005), thus possibly reflecting a post-pandemic effect. Several studies, for instance, demonstrated that the COVID-19 pandemic has deeply disrupted daily life and has created a global context likely to increase ED risk and symptoms, decrease factors that protect against them, and exacerbate barriers to care (e.g., Rodgers et al., 2020). Indeed, this is also in line with recent systematic review data which documented a worsening of ED symptoms among patients with eating disorders (Gao et al., 2022). This data needs attention from the scientific community given the connection between disordered eating, poor psychosocial health, and lower overall health-related quality of life in adolescents (Wu et al., 2019).

Secondly, significant positive associations among the measured variables (i.e., internalized WB, body size dissatisfaction, ED symptoms) and BMI emerged, consistently with the literature supporting strong associations in adolescents (e.g., Troncone et al., 2020) and adults (Innamorati et al., 2017).

Furthermore, gender differences were detected in all the variables examined, highlighting higher scores of ED symptoms, body size dissatisfaction, and internalized WB in female participants coherently with existing literature (e.g., Toselli et al., 2005; Innamorati et al., 2017; Lombardo et al., 2022). The only exception was found in the public WB score, which was higher in males than in females, consistent with previous research using the same instrument among an Italian adult sample (Zagaria et al., 2022). Female students reported also twice the risk of having at least one lifetime weight-related BS experience compared to male students, which is probably related to sociocultural factors, such as the pervasive influence of the thin ideal in feminine beauty standards (Emmer et al., 2020).

Differences emerged also between participants who experienced weight-related BS and participants who did not report it, showing higher scores of public WB (small effect) and internalized WB, ED symptoms, and body size dissatisfaction (large effects) in the first group, thus signaling the importance of weight-related stigmatization in explaining worse psychological health outcomes (Puhl et al., 2017; Puhl and Lessard, 2020). Consistently, this is supported by the results of the mediation model tested only on participants referring overweight: internalized WB partially mediated the relationship between BS by family members and ED symptoms, and fully mediated the relationship between BS by peers and ED symptoms. This model shows that adolescents who perceive themselves as overweight may experience weight-related BS from peers and family members which is associated with higher levels of internalized WB and ED symptoms.

Lastly, as emerged in the second mediation model, public WB seems to have different effects compared to internalized WB, resulting in not being associated with the experience of BS and ED symptoms in adolescent participants with overweight. At first glance, it might seem controversial. However, this might make sense considering that this group (students who report overweight) may have internalized WB by applying negative stereotypes and attitudes to themselves, but have instead developed a greater sensitivity toward other people with impaired weight conditions (i.e., resulting in less negative attitudes toward obesity) and thus presenting a greater heterogeneity that does not support association with BS experiences and ED symptoms.

### 4.1 Strengths and limitations

These findings shed new light not only on the importance of BS experience but also on the role of internalization of WB, which is associated with the worst mental health-related quality of life (as already documented in a sample of women referring overweight and obesity by Zagaria et al., 2023) and may be considered more important than the experience of stigma or weight status alone (Tanas et al., 2022). Of course, since the cross-sectional nature of the study, constitutes one of its main limits, it is difficult to estimate the directionality of the effects, taking into account that they could be bidirectional. However, previous longitudinal results (Puhl et al., 2017) added strong support to the evidence that weight-based teasing in adolescence (and therefore we supposed also WB internalization) can predict higher BMI and obesity in adulthood and ED symptoms in female participants. Consistent with this, clinical reports and excerpts from the life stories of patients seeking help with weight or ED in adulthood support this hypothesis and underscore the importance of longitudinal studies addressing these issues at the national and international levels. A major limitation of the study is related to the self-reported nature of the assessment, especially self-reported height and weight used to calculate BMI, which may be subjected to social desirability and recall biases. Moreover, we used BMI and not BMI-for-age percentile, which can be considered more accurate for the weight classification of children and adolescents, especially when comparing their perception with “standard weight classifications.” However, we were interested in the subjective perception of overweight compared to real nutritional status or weight classification. Therefore, we used this self-reported classification (overweight/not overweight) in both mediation models and not BMI cut-off points to divide our sample. Future studies including both subjective and objectively measured anthropometric parameters, as well as longitudinal designs are needed to further support these preliminary results. Another limitation that should be mentioned, is related to the collection of demographic information among students. For reasons related to time restrictions and the choice not to overload students with a too-long battery of tests, personal data such as socio-demographic conditions related to family income, living conditions, neighborhood or environmental and relational context, as well as sexual orientation were not measured. Indeed, a recent publication suggests that sexual orientation may have an important role in association with weight-related bias (Meneguzzo et al., 2022). Concerning the class/school context, general experiences of bullying and victimization (both actions taken and suffered) have been collected and have been the subject of a recent publication (see Vacca et al., 2023).

### 4.2 Conclusions and future directions

These findings, despite their cross-sectional nature, add an important contribution to the creation of quantitative empirical evidence on the phenomenon of body shaming among adolescent students. A quarter of teenagers reported experiencing body shaming by family members and peers. Its role in explaining eating disorders, both directly and indirectly through the mediation of internalized weight bias, has been first proved and needs to be confirmed by longitudinal results.

These findings underscore the importance of addressing weight-related educational programs and health initiatives, including weight-based anti-bullying interventions, especially among adolescents. There is an urgent need to draw the attention of the scientific and non-scientific community (health professionals, researchers, clinicians, educators, coaches, teachers and parents) to the harmful effect of any form of weight-related prejudice and discrimination. In fact, instead of stimulating healthy and beneficial effects in reducing overweight, obesity or even underweight, they can rather lead to harm and suffering in stigmatized youths (Tanas et al., 2022).

## Data availability statement

The raw data supporting the conclusions of this article will be made available by the authors, without undue reservation.

## Ethics statement

The studies involving humans were approved by Institutional Review Board of the Department of Psychology at Sapienza University of Rome. The studies were conducted in accordance with the local legislation and institutional requirements. Written informed consent for participation in this study was provided by the participants’ legal guardians/next of kin.

## Author contributions

SC: Conceptualization, Data curation, Funding acquisition, Investigation, Methodology, Project administration, Resources, Software, Supervision, Validation, Visualization, Writing – original draft, Writing – review & editing. MV: Conceptualization, Data curation, Investigation, Methodology, Project administration, Resources, Software, Supervision, Validation, Visualization, Writing – review & editing. AZe: Conceptualization, Funding acquisition, Investigation, Methodology, Project administration, Resources, Visualization, Writing – review & editing. AZa: Data curation, Formal analysis, Software, Visualization, Writing – review & editing. CL: Conceptualization, Funding acquisition, Investigation, Methodology, Project administration, Resources, Supervision, Validation, Visualization, Writing – review & editing.

## Footnotes

^1^ https://www.qualtrics.com/
